# Effect of in‐office bleaching on the color stability of bulk‐fill resin composites immersed in staining beverages

**DOI:** 10.1111/eos.70097

**Published:** 2026-04-29

**Authors:** Matheus Kury, Thaís T. Feldens, Márcia V. G. B. Queiroz, Mayara A. P. Oliveira, Luciano S. Gonçalves, Maria Carolina G. Erhardt

**Affiliations:** ^1^ Dental Research Division, Faculty of Dentistry Universidade Paulista (UNIP) São Paulo Brazil; ^2^ Conservative Dentistry Department, Faculty of Dentistry Federal University of Rio Grande do Sul (UFRGS) Porto Alegre Rio Grande do Sul Brazil

**Keywords:** bulk‐fill resin composite, color stability, staining, tooth bleaching

## Abstract

This study evaluated the impact of in‐office bleaching on color stability of bulk‐fill composites submitted to staining. Bulk‐fill (Aura, Opus, and Filtek One) and nanofilled (Filtek Z350) composite specimens were submitted to the following: control (CT); bleaching and immersion in distilled water (BL); and bleaching with daily 15‐min immersion in coffee (BL + CF), cola‐based drink (BL + CO), or red wine (BL + RW) among sessions. A spectrophotometer determined Δ*E*_00_, ΔWI_D_, and coordinates. Degree of conversion (DC) and topography of the composites were assessed using Fourier‐transform infrared spectroscopy in attenuated total reflectance (FTIR‐ATR) and scanning electron microscopy (SEM), respectively. Quantitative data were submitted to statistical tests according to the factors (*α* = 5%). In BL, Opus and Aura displayed significantly higher Δ*E*_00_ and ΔWI_D_ than One. Compared to BL, only BL + CF significantly decreased the ΔWI_D_ and Δ*L* for Aura, but all staining immersions decreased ΔWI_D_ and Δ*L* for Opus. One and Z350 were not impacted by treatments, except for BL + CF, which significantly decreased ΔWI_D_ and Δ*L*. SEM revealed influence of cycling on composites’ surface, but DC did not determine their staining resistance. In‐office bleaching compromised bulk‐fill composite color stability, with Aura and Opus showing unacceptable changes, whereas Filtek One and Z350XT remained acceptable. Staining challenges altered Opus stability, and coffee immersion affected colorimetric outcomes across all composites after bleaching.

## INTRODUCTION

Esthetic demands in Dentistry have grown considerably in recent years, driven by patients’ increasing concern with self‐image and social perception [1, 2, 3]. Resin composites are among the most widely used restorative materials due to their optical properties, conservative nature, and clinical versatility [4]. However, the long‐term esthetic performance of these materials can be compromised by discoloration [5, 6]. Chemical discoloration of resin composites may be attributed to the oxidation in the amine accelerator, structure of the polymer matrix, and unreacted methacrylate groups, which are influenced by the degree of monomer conversion [7, 8]. In addition, the filler–matrix is probably the weakest part of the resin composite. Even with the improvement in the properties of the silanized resin composite, substances present in the oral cavity, such as water, beverages, acids, and enzymes, can diffuse into the composite, potentially accelerating the hydrolysis of the filler's coupling agent [8] and facilitating the degradation of the composite and the penetration of pigments.

Bulk‐fill resin composites were introduced to allow insertion in increments up to 4–5 mm, owing to increased translucency, optimized photo initiator systems, and modified monomer chemistry [9, 10, 11]. Although several studies have investigated their mechanical properties, marginal adaptation, and depth of cure, there is limited information regarding their long‐term optical stability, particularly when subjected to bleaching procedures [6, 12]. In this context, tooth bleaching with hydrogen peroxide is a conservative and widely accepted esthetic treatment, capable of improving dental color without significant alteration to enamel and dentin [13]. Nevertheless, evidence indicates that bleaching agents may negatively affect restorative materials, increasing surface roughness and altering their susceptibility to staining [14].

Considering the growing use of bulk‐fill composites in clinical practice, understanding the interaction between bleaching agents, staining beverages, and bulk‐fill resins is essential to support evidence‐based restorative strategies. A recent scoping review indicated that bulk‐fill materials do not behave uniformly with respect to color stability because their susceptibility to staining varies according to differences in monomer composition, degree of conversion (DC), hydrophilicity, water sorption characteristics, filler loading and morphology, quality of filler–matrix integration, and the type of photoinitiator system employed [6]. These compositional and structural variations have been associated with divergent staining outcomes among commercially available bulk‐fills, with some materials showing greater color change in pigmented solutions, whereas others demonstrate relatively higher stability [15, 16, 17, 18]. This variability reinforces the need to investigate individual bulk‐fill formulations, rather than assuming a homogeneous behavior across the category.

Therefore, the aim of this study was to evaluate the effect of in‐office bleaching with 35% hydrogen peroxide on the color stability of three bulk‐fill resin composites with different compositions immersed in common staining beverages (i.e., red wine, cola‐based soft drink, and brewed coffee). The null hypotheses were that (1) bleaching would not influence the color stability of the bulk‐fill composites, (2) the type of bulk‐fill composite would not play a role in the colorimetric outcomes, and (3) staining immersion protocols would not impact the effect of bleaching on the materials. Additionally, this study aimed to determine whether the cycling protocols produced changes in the surface topography of the composites and to assess whether the DC differed among the bulk‐fill materials.

## MATERIAL AND METHODS

### Experimental design

Cylindrical resin composite specimens were randomly assigned according to the following factors: resin composite and treatment. The resin composites evaluated were Aura bulk‐fill (AU—SDI), Opus bulk‐fill (OP—FGM), Filtek One bulk‐fill (ONE—Solventum), and Filtek Z350XT (Z350—Solventum), as described in Table 1. The treatment conditions consisted of control (CT—no bleaching and no staining), bleaching only (BL), bleaching with immersion in coffee (BL + CF), bleaching with immersion in cola‐based soft drink (BL + CO), and bleaching with immersion in red wine (BL + RW).

**TABLE 1:** Resin composites used in this study and their compositions.

Material	Manufacturer	Composition
**Aura Bulk Fill**	SDI	UDMA, Bis‐EMA, BisGMA, TEGDMA, barium alumino‐borosilicate glass, and silica
**Opus Bulk Fill APS**	FGM	Urethane dimethacrylate monomers, stabilizers, photoinitiator system (APS) and co‐initiators, inorganic filler of silanized silicon dioxide (silica), stabilizers, and pigments
**Filtek One Bulk Fill Restorative**	Solventum	Silanized treated ceramic, aromatic urethane dimethacrylate, diurethane dimethacrylate (UDMA), ytterbium fluoride (YbF_3_), silane‐treated silica, 1,12‐dodecane dimethacrylate (DDDMA), silanized treated zirconia, and water
**Filtek Z350 XT**	Solventum	Treated silanized ceramic, silane‐treated silica, urethane dimethacrylate (UDMA), bisphenol A polyethylene glycol diether dimethacrylate, bisphenol A diglycidyl ether dimethacrylate (BisGMA), zirconia ceramic (66402‐68‐4) surface modified with 3‐methacryloxypropyltrimethoxysilane (2530‐85‐0), bulk‐fill material, polyethylene glycol dimethacrylate, and triethylene glycol dimethacrylate

The specimens were submitted to three sessions of in‐office bleaching with high‐concentration hydrogen peroxide, being stored in different types of staining solutions between the sessions. Blinding was implemented by coding the specimens, ensuring that the operator was unaware of group allocation during bleaching, staining procedures, and colorimetric evaluations. Color coordinates (L*, a*, and b*) of the specimens were measured to calculate color (Δ*E*_00_) and whiteness (ΔWI_D_) changes. Moreover, a morphology evaluation of the composites’ surface after all protocols was conducted by means of scanning electron microscopy (SEM). In addition, DC of the four composites was assessed using Fourier‐transformed infrared spectroscopy (FTIR).

### Specimens’ preparation

Twenty cylindrical specimens (6 mm diameter × 5 mm height) were prepared in shade A2 for each composite resin tested, except for Aura SDI, which is available only in a universal shade. All the resin composite materials used are described in Table 1.

Polyvynilsiloxane molds (Zetaplus, Zhermack) and polyester strips on glass slabs were used to standardize specimen fabrication. Composite resins were inserted in a single increment and covered with a polyester strip. The conventional nanofilled composite was incrementally built up. Specimens were light‐cured for 20 s using an LED unit (Elipar DeepCure‐L, 3M, Brazil; 1470 mW/cm^2^, 430–480 nm) and stored in distilled water at 37°C for 48 h to ensure complete polymerization. Specimens were randomly assigned to experimental groups (*n* = 4/group) using random.org. The sample size was determined using G*Power for anova (fixed effects, special, main effects, and interactions). The level of significance was set at 0.05, and the power of the test was 0.8, alongside an effect size of 0.40 and degrees of freedom ranging from 3 to 4. According to the factors and levels of study, the following groups in Table 2 were determined.

**TABLE 2:** Group distribution of the present study, taking into consideration the factors under study and levels.

Composite resin	No bleaching (CT)	Only bleaching (BL)	Bleaching + coffee (BL + CF)	Bleaching + cola‐based soft drink (BL + CO)	Bleaching + red wine (BL + RW)
Aura Bulk (AU)	AU/CT	AU/BL	AU/BL + CF	AU/BL + CO	AU/BL + RW
Opus Bulk (OP)	OP/CT	OP/BL	OP/BL + CF	OP/BL + CO	OP/BL + RW
Filtek One Bulk (ONE)	ONE/CT	ONE/BL	ONE/BL + CF	ONE/BL + CO	ONE/BL + RW
Filtek Z350 (Z350)	Z350/CT	Z350/BL	Z350/BL + CF	Z350/BL + CO	Z350/BL + RW

### Bleaching and staining procedures

The specimens were submitted to three in‐office bleaching sessions with 35% hydrogen peroxide gel (Whiteness HP, FGM). The gel was applied in a standardized layer thickness for 45 min, without renewal [19]. The sessions were repeated every 96 h. In the intervals, the specimens were stored in distilled water. The specimens were also daily immersed in different staining solutions for 15 min. On the days when bleaching has occurred, staining immersion took place 2 h after bleaching exposure. The time of daily immersion was adapted from previous studies [20, 21, 22].

**FIGURE 1:**
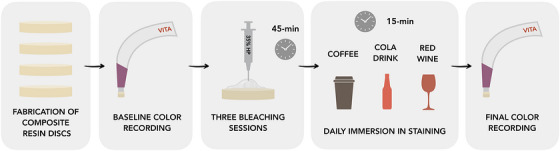
Diagram of the cycling protocols before and after color measurements.

The following staining types were used as follows: Chalise sweet red wine (Salton Winery), cola‐based soft drink (Coca‐Cola), and traditional roasted and ground coffee (Melitta Brasil). The coffee solution was prepared according to the manufacturer's instructions, with a ratio of 4 tablespoons of coffee (80 g) to 1 L of hot water. The specimens were immersed in the staining solutions for 15 min daily for 8 d. After the staining procedures were completed, the specimens were washed in running water, immersed again in distilled water, and kept in an oven at 37°C, being removed only at the time of color measurement. The diagram presented in Figure 1 illustrated the flow chart of the present research regarding the bleaching and staining protocols before and after color measurements.

### Colorimetric evaluation

Color measurements were performed using a calibrated VITA Easyshade V spectrophotometer (Vita, Zahnfabrik). Measurements were taken with the specimens positioned on a white opaque background. Each specimen was measured three times per time point, and the mean CIE Lab* values were recorded. The color was measured always at the same side, which was used to also light curing the specimens. Color coordinates were evaluated at baseline (after 48 h in distilled water) and 2 h after the final bleaching session and immersion in distilled water. Coordinate changes were calculated (Δ*L*, Δ*a*, and Δ*b*). The color change was calculated using the Δ*E*_00_ and ΔWI_D_ parameters. The color variation was evaluated using the CIEDE2000 formula (Δ*E*_00_):

$$\Delta E_{00}=\sqrt{{\left(\frac{\Delta L^{\prime }}{K_{L}SL}\right)}^{2}+{\left(\frac{\Delta C^{\prime }}{K_{C}SC}\right)}^{2}+{\left(\frac{\Delta H^{\prime }}{K_{H}SH}\right)}^{2}+RT\left(\frac{\Delta C^{\prime }}{K_{C}SC}\right)\;\left(\frac{\Delta H^{\prime }}{K_{H}SH}\right)}$$

The variation in the whiteness index was calculated using the following formula:

$$WI_{D}=\left[\left(0.511\times L^{*}\right)-\left(2.324\times a^{*}\right)-\left(1.100\times b^{*}\right)\right]$$

The Δ*E*_00_ values were assessed using the perception threshold (PT) and the acceptance threshold (AT), with values of 0.81 and 1.8 U, respectively. Similarly, the whiteness index changes (ΔWI_D_) were compared against PT and AT thresholds of 0.7 and 2.6, respectively [23].

### Scanning electron microscopy (SEM) analysis

To evaluate whether the experimental conditions produced detectable alterations in the surface morphology of the resin composites, two additional specimens per group (*n* = 2) were prepared following the same procedures described previously and were randomly allocated to the corresponding experimental groups. After the completion of the treatments assigned to each group, the specimens were subjected to an ultrasonic bath for 15 min to remove any loosely attached debris. The samples were then stored in a desiccator containing silica gel at 37°C for dehydration. Before preparing the specimens for SEM, images were acquired with a digital camera at controlled conditions of illumination, distance, and operator.

Subsequently, the dried specimens were sputter‐coated with a thin layer of gold using a metal coater (MED 010). Surface morphology was examined under a scanning electron microscope (JEOL JSM‐6460LV) at an accelerating voltage of 15 kV. For each specimen, three representative micrographs were acquired from randomly selected surface regions at 2000× magnification.

### Degree of conversion

The DC of the resin composites was evaluated using FTIR spectroscopy in attenuated total reflectance (ATR) mode (Nicolet i50, Thermo Fisher Scientific). For this analysis, three additional specimens were prepared for each composite resin. Specimens of Filtek Z350XT were prepared at 2 mm thickness as they were previously incrementally built up, whereas the bulk‐fill composites (Aura Bulk Fill, Opus Bulk Fill APS, and Filtek One Bulk Fill) evaluation was performed at 5 mm thick. For this section, the specimens were not submitted to bleaching and staining challenges.

Unpolymerized material from each composite was placed directly onto the ATR crystal to obtain the uncured baseline spectrum. The corresponding cured spectra of the top portion of the specimens were collected after a light activation of the standardized specimens. The top portion was assessed as the previous measurements (color and SEM analysis) were conducted also in the top side of the composites. The photopolymerization parameters were identical to those aforementioned, including light‐curing unit, irradiance, exposure time, and positioning of the curing tip. All spectra were acquired at a resolution of 4 cm^−1^ with 32 scans. The DC was calculated by monitoring the reduction in the aliphatic C = C stretching peak at 1637 cm^−1^, using the aromatic C–C peak at 1608 cm^−1^ as an internal reference for AU and OP composites. The reference peaks for ONE and Z350 were 1720 and 1455 cm^−1^, respectively. The DC (%) was determined using the following formula:

$$\mathrm{DC}\;\left(\%\right)=\left[1-\frac{{\left(A_{1637}/A_{\mathrm{internal}\;\mathrm{reference}\;\mathrm{peak}}\right)}_{\mathrm{cured}}}{{\left(A_{1637}/A_{\mathrm{internal}\;\mathrm{reference}\;\mathrm{peak}}\right)}_{\mathrm{uncured}}}\right]\times 100$$

where *A*_1637_ and Ainternal reference peaks correspond to the absorbance intensities at those respective wavenumbers.

### Statistical analyses

Data were explored in terms of normality (Shapiro–Wilk) and homoscedasticity (Brown–Forsyth). Baseline L*, a*, and b* coordinate values were evaluated using generalized linear models and Bonferroni's tests. Δ*E*_00_, ΔWI_D_, Δ*L*, and Δ*b* values were submitted to two‐way anova and Bonferroni's tests. DC values were tested using one‐way anova and Tukey's tests. As Δ*a* values did not follow the assumptions of normality, its values were submitted to two‐way anova on the Ranks and Student–Newman–Keuls tests. The tests were performed using sigmaplot statistical software (Grafiti LLC), taking into consideration a 0.05 level of significance (*α* = 5%).

## RESULTS

**FIGURE 2:**
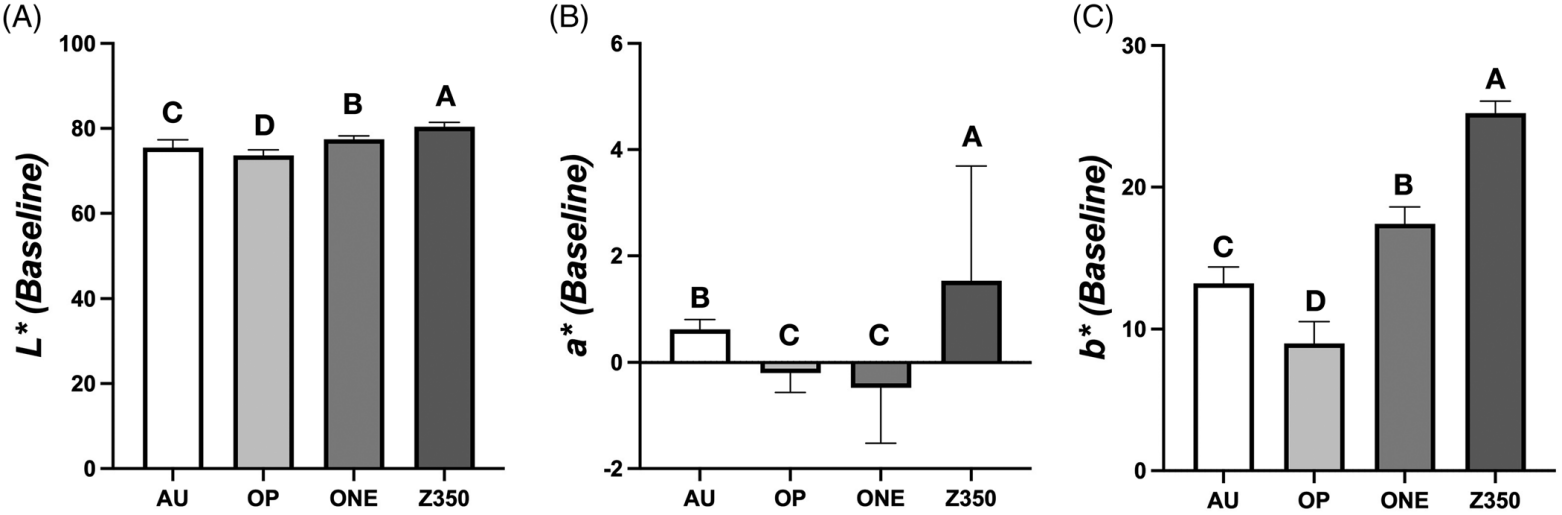
Baseline mean and standard deviation L* (A), a* (B), and b* (C) values of the groups. Different uppercase letters indicate significant differences among the resin composites within the same treatment, considering generalized linear models and Bonferroni tests (*α* = 5%).

Figure 2 depicts the baseline mean and standard deviations of the Euclidean coordinates. For the L* parameter, statistically significant differences were found among all materials. Z350 showed the highest L*, followed by ONE, AU, and OP. For the a* parameter, Z350 presented significantly higher values than the other composites. AU showed intermediate values, whereas OP and ONE did not differ from each other. For the b* parameter, statistically significant differences were also observed among all groups. Z350 exhibited the highest mean value, followed by ONE, AU, and OP.

**FIGURE 3:**
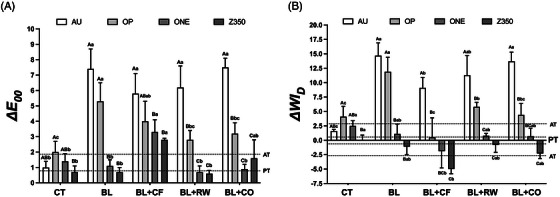
Mean and standard deviation Δ*E*_00_ (A) and ΔWI_D_ (B) values of the groups comparing *baseline* to *after the last bleaching session* time points. Different uppercase letters indicate significant differences among the resin composites within the same treatment. Different lowercase letters indicate significant differences within the same resin composite among the different treatments, considering two‐way anova and Bonferroni tests (*α* = 5%). PT = percpetibility threshold (Δ*E*_00_ = 0.8; ΔWI_D_ = 0.7) and AT = acceptability thresholds (Δ*E*_00_ = 1.8; ΔWI_D_ = 2.7).

Figure 3 illustrates the mean and standard deviation values of Δ*E*_00_ and ΔWI_D_. Two‐way anova revealed that the factors “resin composite” and “treatment” and their interaction affected the outcomes (*p* < 0.001). In CT, OP exhibited significantly higher Δ*E*_00_ and ΔWI_D_ than Z350, whereas AU and ONE presented intermediate values, without statistical differences to OP and Z350. After only BL, AU and OP showed significantly higher Δ*E*_00_ and ΔWI_D_ than ONE and Z350. After BL + CF, only AU demonstrated significantly higher Δ*E*_00_ than ONE and Z350. OP did not show differences to all the comparator composites. Under BL + CO and BL + RW, AU reached the highest Δ*E*_00_ and ΔWI_D_ (*p* < 0.05), followed by OP, which was also higher than ONE and Z350 in terms of Δ*E*_00_ (*p* < 0.05). Across all treatments, no differences were observed between ONE and Z350 (*p* > 0.05).

No differences in Δ*E*_00_ were detected among bleached groups for AU, regardless of the staining (*p* > 0.05), but it showed lower ΔWI_D_ when comparing BL + CF to BL (*p* < 0.05). The lowest Δ*E*_00_ and ΔWI_D_ were observed for AU in CT (*p* < 0.05). Δ*E*_00_ and ΔWI_D_ values exceeded both PT and AT across all treatments, except in CT.

For OP, the highest Δ*E*_00_ and ΔWI_D_ were recorded after BL, and ΔWI_D_ significantly decreased after all staining immersions. BL + CF rendered lower ΔWI_D_ than BL + CO (*p* < 0.05), but BL + RW promoted similar Δ*E*_00_ and ΔWI_D_ to them. All treatments resulted in Δ*E*_00_ and ΔWI_D_ above both PT and AT, except for BL + CF.

For ONE, BL + CF during bleaching resulted in the highest Δ*E*_00_ (*p* < 0.05), whereas no significant differences were observed among the other treatments. In terms of ΔWI_D_, BL + CF reached a significant difference to CT, with a negative mean ΔWI_D_. In Z350, significantly higher Δ*E*_00_ values were found after BL + CF compared with all other treatments, except BL + RW. All bleached groups presented negative mean ΔWI_D_, but only BL + CF had a statistical difference to CT in Z350. For both ONE and Z350 after BL + CF, Δ*E*_00_ surpassed PT and AT, whereas only Z350 had overcome the AT for ΔWI_D_.

**FIGURE 4:**
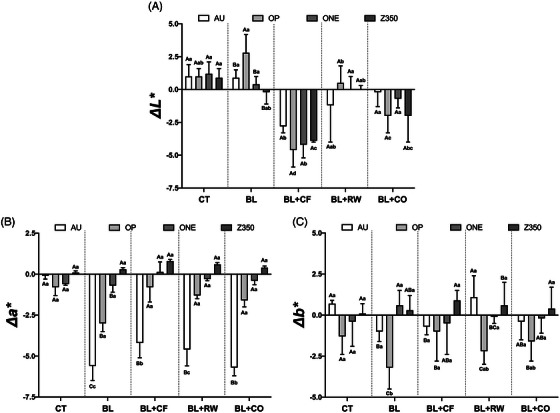
Mean and standard deviation ΔL* (A), Δa* (B), and Δb* (C) values of the groups comparing *baseline* to *after the last bleaching session* time points. Different uppercase letters indicate significant differences among the resin composites within the same treatment. Different lowercase letters indicate significant differences within the same resin composite among different treatments, taking into consideration two‐way anova and Bonferroni tests, or two‐way anova on Ranks and Student–Newman–Keuls tests (*α* = 5%).

Figure 4 demonstrates the Δ*L*, Δ*a*, and Δ*b* results. No significant differences were detected in CT groups for all coordinate changes. Under only BL, OP presented the highest Δ*L* and Δ*b*, whereas AU exhibited the highest Δ*a* (*p* < 0.05). Among BL + CF, no differences were detected for Δ*L* (*p* > 0.05). Among BL + CO, AU presented Δ*a* and Δ*b* higher than all other materials (*p* < 0.05). ONE and Z350 promoted intermediate Δ*b*. In BL + RW, AU also reached the highest Δ*a* (*p* < 0.05), but with similar Δ*b* to the others. Overall, as seen in Figure 4, BL + CF resulted in significantly higher Δ*L* than all other treatments, but all composites treated with BL + CF and BL + RW had negative Δ*L*. AU composite caused higher Δ*a* than all the other composites among treatments (*p* < 0.05). No significant Δ*a* and Δ*b* differences were detected among AU, ONE, and Z350 across all treatments. BL led OP composite to have significantly higher Δ*b* than BL + CF and BL + RW.

**FIGURE 5:**
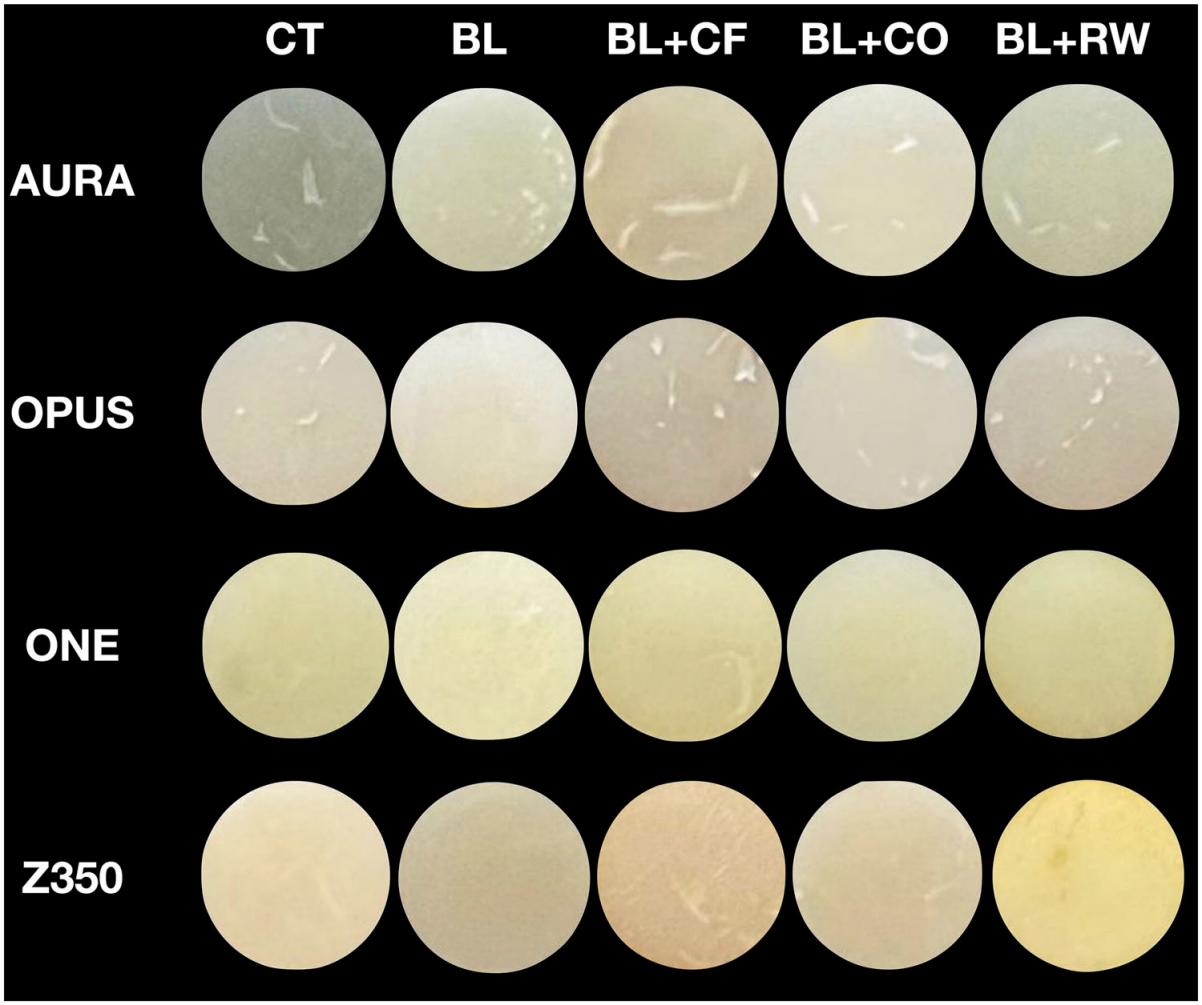
Digital photography of the composites under all conditions. At baseline, AU appeared more translucent, whereas ONE and Z350 showed a more yellowish tone. After bleaching, only AU and OP appeared visibly lighter. CF apparently caused darkening in all materials, RW had a more pronounce effect on Z350, and CO seems to have preserved the bleaching effect.

Figure 5 illustrates the digital photography taken before the SEM preparation. At baseline, all composites exhibited visibly distinct appearances, with AU showing a more translucent aspect, whereas ONE and Z350 appeared comparatively more yellowish. After bleaching, only AU and OP demonstrated a perceptible whitening effect. When evaluating the post‐bleaching pigmentation conditions, CF was the only staining solution that consistently darkened all resins across all materials and conditions. The effect of RW was more apparent for OP and Z350, whereas CO generally preserved the whitening effect, showing minimal visual darkening in all groups.

**FIGURE 6:**
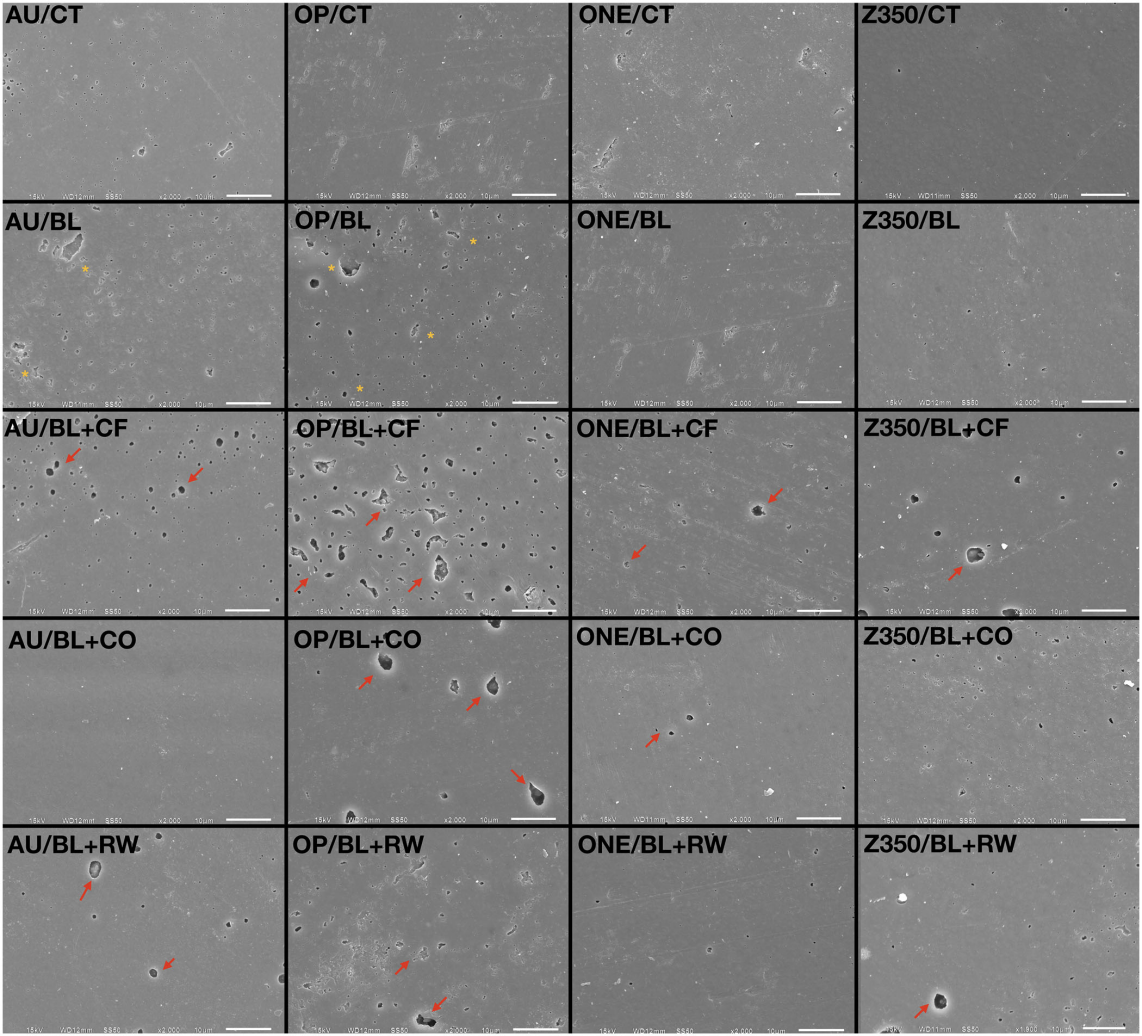
Representative scanning electron microscopy (SEM) micrographs for AU, OP, ONE, and Z350 resin composites in control (CT), bleached (BL), and bleached + stained groups (BL + CF, BL + CO, and BL + RW). The yellow asterisks indicate regions where bleaching alone produced visible surface alterations compared with the corresponding control groups (OP/CT and AU/CT). The red arrows highlight voids and defects suggestive of filler particle dislodgement, which were more evident in groups exposed to coffee (CO) and in several Opus (OP) conditions. Scale bar = 10 *µ*m.

The SEM micrographs (Figure 6) illustrate the surface morphology of the resin composites across all experimental conditions. In CT condition, specimens of ONE/CT and OP/CT already exhibited areas of surface irregularity and localized loss of smoothness after storage in distilled water alone, whereas AU/CT and Z350/CT showed comparatively more uniform surfaces. In BL condition, additional changes were observed for OP/BL and AU/BL, indicated by the yellow asterisks in the images. These regions display subtle alterations relative to their respective controls (OP/CT and AU/CT), suggesting that bleaching alone produced detectable modifications in surface texture for these specific materials.

In the groups exposed to bleaching followed by staining solutions (BL + CF, BL + CO, and BL + RW), the red arrows suggest the presence of multiple voids or surface defects consistent with filler particle dislodgement, leaving empty spaces within the resin matrix. This pattern was most pronounced in all groups stored in coffee (CO) and in several conditions involving the OP resin, where the number and size of voids appeared greater than in the other materials. These features were visibly more frequent in OP/BL + CF, OP/BL + CO, and OP/BL + RW, with minor prevalence in AU/BL + RW, ONE/BL + CO, and Z350/BL + RW.

Table 3 summarizes the DC measured from the top surface of the composite resins evaluated in this study. Although numerical variations were observed among the materials, no statistically significant differences were detected in DC values across the composite resins under the curing conditions tested.

**TABLE 3:** Degree of conversion acquired from the top portion of the composited used in this study.

Composite resin	DC (%)
Aura bulk (AU)	56.6 (3.4)
Opus bulk (OP)	54.7 (3.4)
Filtek one bulk (ONE)	58.2 (1.5)
Filtek Z350 (Z350)	53.3 (4.0)

*Note*: No significant differences were detected among the groups (anova/*p* = 0.504).

Abbreviation: DC, degree of conversion.

## DISCUSSION

The present findings revealed that in‐office bleaching alone significantly influenced the color change and whiteness index of at least two bulk‐fill composites compared to their corresponding control groups. Therefore, the first null hypothesis that bleaching alone would not impact the color stability of the materials was rejected. Aura and Opus bulk‐fill composites showed Δ*E*_00_ and ΔWI_D_ values exceeding the acceptability threshold, suggesting that these restorations may require polishing or replacement after bleaching procedures. It is worth highlighting that the direction of the color change indicated a transition toward a whiter and more light‐reflective appearance, as denoted by positive ΔWI_D_. On the other hand, Filtek One exhibited Δ*E*_00_ and ΔWI_D_ without statistical differences compared to the nanofilled composite (Filtek Z350XT), which did not surpass the acceptability thresholds and did not differ from their corresponding control groups.

Previous studies have demonstrated that hydrogen peroxide‐based bleaching agents may induce alterations on resin composites, mainly by increasing surface roughness, reducing microhardness, and promoting chemical degradation of the resin matrix due to the oxidative potential of free radicals [24, 25, 26]. Such changes have been attributed to the diffusion of peroxide molecules through the organic phase, which may lead to polymer degradation and filler–matrix debonding. In the context of bulk‐fill composites, few studies have also observed significant modifications in color parameters and surface morphology following exposure to high‐concentration bleaching gels [10, 27, 28, 29].

According to the present results, the impact of bleaching on colorimetric changes was significantly influenced by the type of the bulk‐fill composite, rejecting the second null hypothesis. Indeed, groups with AU and OP composites after bleaching only (BL) showed more pronounced morphological changes when compared to their respective controls. In a previous study, Schuster *et al.* showed that bleaching with a commercial 40% HP gel provoked the elution of important monomers from the organic matrix compared to unbleached bulk‐fill composite [30]. In this context, the localized surface alterations observed for AU/BL and OP/BL may be related to compositional characteristics that render these materials more susceptible to peroxide‐induced degradation. Bulk‐fill composites containing more hydrophilic monomers or a higher proportion of organic matrix are known to exhibit greater water sorption and matrix plasticization, which may facilitate the oxidative action of hydrogen peroxide and the hydrolysis of the silane‐filler interface [31, 32].

In other words, the greater susceptibility of Aura and Opus might be associated with the proportion of their monomers (BisGMA, UDMA, BisEMA, and TEGDMA) and glass/silica fillers, which could be more prone to oxidative degradation and filler‐matrix debonding [33]. In this sense, the high filler content described for both Aura (72.7 wt%) [34] and Opus (79.0 wt%) [35] may not correspond to a high filler content in volume ratio [36], thereby potentially increasing the interaction with hydrogen peroxide due to greater matrix exposure at the surface. In addition, once monomer leaching may occur, metal oxides used as pigments (e.g., iron and titanium oxides) [37] may become more exposed and subsequently degraded, further contributing to color alteration and positive ΔWI_D_. However, this interpretation should be considered with caution, as direct evidence was not assessed in the present study.

Filtek One, in turn, incorporates high‐molecular‐weight and more hydrophobic monomers that could reduce monomer leaching, whereas Filtek Z350XT contains a dense silica/zirconia nanofiller network that could limit peroxide diffusion [8]. Despite compositional differences, both materials may present a more stable resin–filler interface and lower permeability to bleaching agents, which could explain their similar colorimetric stability.

When it comes to staining immersion cycling, it is noticeable that the different solutions significantly impacted the Δ*E*_00_ and ΔWI_D_ reached by Aura and Opus that underwent bleaching intermittently, rejecting the third null hypothesis. Nevertheless, the susceptibility to staining was more pronounced for Opus, whose ΔWI_D_ significantly decreased under all staining types compared to BL only, whereas Aura was only statistically affected by coffee staining. The coordinate changes illustrated that coffee was the only solution that significantly decreased the Δ*L* in Aura groups, confirming a reduction in luminosity. Nonetheless, it is important to emphasize that all AU and OP groups under immersion still exhibited positive ΔWI_D_ values, indicating that bleaching remained the predominant effect throughout the cycling protocol.

The selective effect of coffee on Aura may be explained by the chemical nature of this pigment, which consists of small and polar molecules capable of diffusing more easily through microstructural defects or increased surface roughness induced by bleaching [8, 11]. Importantly, even Filtek One and Z350 submitted to coffee during bleaching had their Δ*E*_00_, ΔWI_D_, and Δ*L* significantly affected, indicating a darker appearance. From a clinical perspective, this finding is relevant, as patients commonly consume coffee during bleaching treatments [38].

In addition, the pigments in red wine are mainly anthocyanins and tannins, which have a higher molecular weight but remain highly chromogenic [39], as seen by Z350 submitted to RW. The ethanol content may further contribute by increasing matrix permeability [40]. On the other hand, the color of cola is derived from artificial colorants, which tend to produce less pronounced changes despite the low pH [41].

Finally, although previous literature suggests that the DC may influence staining susceptibility [6], the present findings did not show significant differences among the materials. The DC (%) values obtained in the present study are consistent with previous reports in the literature, including measurements taken from the top surface of composite restorations [42, 43, 44, 45]. The fact that Z350/BL + CF exhibited the greatest darkening among all groups, whereas the Aura groups showed the highest whitening after bleaching exposure, further reinforces the lack of influence of DC on the interaction with either bleaching or staining solutions. This reinforces that color changes are likely governed by other factors, such as matrix composition, hydrophilicity, and interfacial stability rather than DC alone.

The present in vitro design carries limitations, as it cannot fully simulate the complex oral environment, where saliva, pellicle, and mechanical wear influence the interaction between bleaching agents and composites. Staining protocols were adapted from previous studies but do not capture the variability of real consumption habits. Moreover, only a limited number of bulk‐fill composites were tested, restricting extrapolation to other materials. Future studies should explore long‐term in situ and clinical designs, include a wider range of composites, and evaluate protective or restorative strategies under concomitant bleaching and staining conditions.

## CONCLUSION

Within the limitations of the present study, the following conclusions could be drawn:

- In‐office bleaching with high‐concentrated hydrogen peroxide gel significantly compromised the color stability of bulk‐fill composites in a material‐dependent manner: Aura and Opus showed unacceptable Δ*E*_00_ and positive ΔWI_D_ values compared to unbleached groups, whereas Filtek One (bulk‐fill) and Filtek Z350XT (nanofilled) maintained acceptable values.
- The positive ΔWI_D_ reached by bleaching on Opus was significantly reduced by all types of staining, but staining with coffee also impacted the Δ*E*_00_ and/or ΔWI_D_ reached by all the other composites after bleaching.
- Additional analyses showed that the cycling protocols modified the surface topography of the bulk‐fill composites, suggesting that material composition—and not the DC—played a more substantial role in determining their color stability.

## AUTHOR CONTRIBUTIONS

**Conceptualization**: Maria Carolina G. Erhardt and Thaís T. Feldens. **Formal analysis**: Matheus Kury and Maria Carolina G. Erhardt. **Investigation**: Thaís T. Feldens, Márcia V. G. B. Queiroz, and Mayara A. P. Oliveira. **Methodology**: Thaís T. Feldens and Márcia V. G. B. Queiroz. **Writing—original draft**: Matheus Kury, Márcia V. G. B. Queiroz, and Mayara A. P. Oliveira. **Writing—review and editing**: Maria Carolina G. Erhardt and Luciano S. Gonçalves.

## CONFLICT OF INTEREST STATEMENT

The authors declare no conflicts of interest.

## Data Availability

The data that support the findings of this study are available from the corresponding author upon reasonable request.

## References

[1] Ghorbani Z , Esmaeili S , Shahbazi S , Jarrahzadeh M , Madihi S . Self‐esteem and its influence on the inclination toward esthetic dental treatments: a cross‐sectional study. BMC Psychol. 2025;13(1):140. 10.1186/s40359-025-02423-7 39972400 PMC11840998

[2] Maran BM , Bersezio C , Martin J , Favoreto MW , Rezende M , Vallejo‐Izquierdo L , et al. The influence of dental bleaching on patient's quality of life: a multistudy analysis of aesthetic and psychosocial outcomes. J Dent. 2024;151:105397. 10.1016/j.jdent.2024.105397 39378962

[3] Goettems ML , Fernandez MDS , Donassollo TA , Donassollo SH , Demarco FF . Impact of tooth bleaching on oral health‐related quality of life in adults: a triple‐blind randomised clinical trial. J Dent. 2021;105:103564. 10.1016/j.jdent.2020.103564 33359042

[4] Pizzolotto L , Moraes RR . Resin composites in posterior teeth: clinical performance and direct restorative techniques. Dent J (Basel). 2022;10(12):222. 10.3390/dj10120222 36547038 PMC9777426

[5] Kareem ASA , Abdel‐Fattah WM , El Gayar MIL . Evaluation of color stability and surface roughness of smart monochromatic resin composite in comparison to universal resin composites after immersion in staining solutions. BMC Oral Health. 2025;25(1):1211. 10.1186/s12903-025-06555-5 40684165 PMC12276654

[6] Paolone G , Mandurino M , Scotti N , Cantatore G , Blatz MB . Color stability of bulk‐fill compared to conventional resin‐based composites: a scoping review. J Esthet Restor Dent. 2023;35(4):657–76. 10.1111/jerd.13017 36789480

[7] Szczesio‐Wlodarczyk A , Sokolowski J , Kleczewska J , Bociong K . Ageing of dental composites based on methacrylate resins‐a critical review of the causes and method of assessment. Polymers (Basel). 2020;12(4):882. 10.3390/polym12040882 32290337 PMC7240588

[8] Cinelli F , Scaminaci Russo D , Nieri M , Giachetti L . Stain susceptibility of composite resins: pigment penetration analysis. Materials (Basel). 2022;15(14):4874. 10.3390/ma15144874 35888342 PMC9320780

[9] Wafaie RA , Ahmed B , Mahmoud SH . Fracture resistance of molars with class II MOD cavities restored with bulk‐fill, no‐cap flowable bulk‐fill, and conventional resin composite restorative systems after 6‐months water storage. BMC Oral Health. 2025;25(1):741. 10.1186/s12903-025-05951-1 40394542 PMC12093850

[10] Sengupta A , Naka O , Mehta SB , Banerji S . The clinical performance of bulk‐fill versus the incremental layered application of direct resin composite restorations: a systematic review. Evid Based Dent. 2023;24(3):143. 10.1038/s41432-023-00905-4 37402908 PMC10516750

[11] Erturk‐Avunduk AT , Cengiz‐Yanardag E , Karakaya I . The effect of bleaching applications on stained bulk‐fill resin composites. BMC Oral Health. 2022;22(1):392. 10.1186/s12903-022-02414-9 36088325 PMC9464385

[12] Popescu AD , Tuculina MJ , Diaconu OA , Gheorghita LM , Nicolicescu C , Cumpătă CN , et al. Effects of dental bleaching agents on the surface roughness of dental restoration materials. Medicina (Kaunas). 2023;59(6):1067. 10.3390/medicina59061067 37374271 PMC10303374

[13] Kury M , Prunes BB , Saraceni CHC , Hilgert LA , Fronza BM , Lima AF . Clinical decision‐making in tooth bleaching based on current evidence: a narrative review. Dent Mater. 2025;41(5):536‐52. 10.1016/j.dental.2025.03.002 40082147

[14] Kayalidere EE , Dorter C . Effects of in‐office bleaching agents on polished and unpolished nanofilled resin composite. J Am Dent Assoc. 2023;154(7):592–600. 10.1016/j.adaj.2023.04.005 37191615

[15] Luque JV , Zubizarreta‐Macho Á , Bartolomé JF , Kois JC , Revilla‐León M . Effect of hydrogen peroxide‐based bleaching agents on the color dimensions and surface roughness of different milled restorative dental materials. Int J Prosthodont. 2023;37:547–58. 10.11607/ijp.8359 39331580

[16] Floriani F , Jurado CA , Madhu N , Lackey MA , FX A‐F , Lopes GC . Color stability of bulk‐fill flowable resin composites after artificial aging. Dent J (Basel). 2024;12(11):350. 10.3390/dj12110350 39590400 PMC11592736

[17] Mailart MC , Rocha RS , Contreras SCM , Torres CRG , Borges AB , Caneppele TMF . Effects of artificial staining on bulk‐filled resin composites. Am J Dent. 2018;31(3):144–8. 30028933

[18] Barutcigil Ç , Barutcigil K , Özarslan MM , Dündar A , Yilmaz B . Color of bulk‐fill composite resin restorative materials. J Esthet Restor Dent. 2018;30(2):E3–E8. 10.1111/jerd.12340 28960790

[19] Kury M , Lins RBE , Resende BA , Picolo MZD , André CB , Cavalli V . The influence of the renewal or the single application of the peroxide gel on the efficacy and tooth sensitivity outcomes of in‐office bleaching‐A systematic review and meta‐analysis. J Esthet Restor Dent. 2022;34(3):490–502. 10.1111/jerd.12827 34623017

[20] Rodrigues CS , Nora BD , Mallmann A , May LG , Jacques LB . Repolishing resin composites after bleaching treatments: effects on color stability and smoothness. Oper Dent. 2019;44(1):54–64. 10.2341/17-107-L 29856701

[21] Ertas E , Güler AU , Yücel AC , Köprülü H , Güler E . Color stability of resin composites after immersion in different drinks. Dent Mater J. 2006;25(2):371–6 16916243

[22] Farghal NS , Abu Shamleh A , Al Hurmuzi O , Mahmoud O . The effects of a carbonated beverage on the optical properties and microhardness of preheated bulk‐fill composite resin restorations. Front Oral Health. 2025;6:1539527. 10.3389/froh.2025.1539527 40529291 PMC12171308

[23] Paravina RD , Pérez MM , Ghinea R . Acceptability and perceptibility thresholds in dentistry: a comprehensive review of clinical and research applications. J Esthet Restor Dent. 2019;31(2):103–12. 10.1111/jerd.12465 30891913

[24] Attin T , Hannig C , Wiegand A , Attin R . Effect of bleaching on restorative materials and restorations—a systematic review. Dent Mat. 2004;20(9), 852–61. 10.1016/j.dental.2004.04.002 15451241

[25] Dos Santos Muniz Mota GM , Kury M , Pereira da Silva Braga Tenório C , Lucisano Botelho do Amaral F , Pedroso Turssi C , Cavalli V . Effects of artificial staining and bleaching protocols on the surface roughness, color, and whiteness changes of an aged nanofilled composite. Front Dent Med. 2020;1:610586. 10.3389/fdmed.2020.610586

[26] Tenório CP , Kury M , Mota GM , Pedroso Turssi C , Lucisano Botelho do Amaral F , Cavalli V . Does the combination of whitening toothpaste and hydrogen peroxide bleaching increase the surface roughness and change the morphology of a nanofilled composite? Braz J Oral Sci. 2024;23:e241938.

[27] Klaric Sever E , Simenc N , Rakic M , Skenderovic H , Sever I , Tarle Z . Effects of bleaching agent on physical and aesthetic properties of restorative materials. Dent Mater J. 2016;35(5):788–95. 10.4012/dmj.2015-443 27725516

[28] Tavares BG , França FM , Basting RT , Turssi CP , Amaral FLB . Effect of bleaching protocols on surface roughness and color change of high‐ and low‐viscosity bulk‐fill composite resins. Acta Odontol Latinoam. 2020;33(2):59–68. 32920607

[29] Araújo RM , Lemes EC , Pachito RF , Feitosa FA . The impact of at home and in‐office bleaching agents on the color stability of bulk‐fill composite resins. Braz Dent Sci. 2019;22(1):94–102.

[30] Schuster L , Reichl FX , Rothmund L , He X , Yang Y , Landuyt KLV , et al. Effect of opalescence(®) bleaching gels on the elution of bulk‐fill composite components. Dent Mater. 2016;32(2):127–35. 10.1016/j.dental.2015.11.033 26719129

[31] Malacarne J , Carvalho RM , de Goes MF , Svizero N , Pashley DH , Tay FR , et al. Water sorption/solubility of dental adhesive resins. Dent Mater. 2006;22(10):973–80. 10.1016/j.dental.2005.11.020 16405987

[32] Gauthier R , Abouelleil H , Boussès Y , Brulat‐Bouchard N , Colon P , Chenal JM , et al. Experimental investigation of dental composites degradation after early water exposure. J Biomech Eng. 2023;145(5):051001. 10.1115/1.4056197 36350265

[33] Barišić ML , Sarajlija H , Klarić E , Knežević A , Sabol I , Pandurić V . Detection of leachable components from conventional and dental bulk‐fill resin composites (high and low viscosity) using liquid chromatography‐tandem mass spectrometry (LC‐MS/MS) method. Polymers (Basel). 2023;15(3):627. 10.3390/polym15030627 36771928 PMC9921113

[34] Jafarpour D , Ferooz R , Ferooz M , Bagheri R . Physical and mechanical properties of bulk‐fill, conventional, and flowable resin composites stored dry and wet. Int J Dent. 2022;2022:7946239. 10.1155/2022/7946239 35186087 PMC8853813

[35] FGM Dental Group . Opus bulk fill APS—technical data sheet. São Paulo: FGM Dental Group; 2023. https://srv01.fgmdentalgroup.com/it/prodotti‐di‐estetica/opus‐bulk‐fill‐aps/

[36] Randolph LD , Palin WM , Leloup G , Leprince JG . Filler characteristics of modern dental resin composites and their influence on physico‐mechanical properties. Dent Mater. 2016;32(12):1586–99. 10.1016/j.dental.2016.09.034 27720423

[37] Haas K , Azhar G , Wood DJ , Moharamzadeh K , Noort R . The effects of different opacifiers on the translucency of experimental dental composite resins. Dent Mater. 2017;33(8):e310–e6. 10.1016/j.dental.2017.04.026 28535952

[38] Rossi B , Massarenti AHM , Pizzanelli GG , Queiroz MVGB , Santos FMD , Kury M , et al. Post‐curing effects on mechanical properties staining resistance of a 3D‐printed resin versus a direct composite for definitive restorations. Eur J Oral Sci. 2026:e70093. 10.1111/eos.70093 42003561 PMC13377921

[39] Nel AP . Tannins and anthocyanins: from their origin to wine analysis. S Afr J Enol Vitic. 2018;39(1):1–14.

[40] de Andrade ICGB , Basting RT , Rodrigues JA , Amaral FLB , Turssi CP , França FMG . Microhardness and color monitoring of nanofilled resin composite after bleaching and staining. Eur J Dent. 2014;8(2):160–5. 10.4103/1305-7456.130586 24966764 PMC4054044

[41] Manojlovic D , Lenhardt L , Milićević B , Antonov M , Miletic V , Dramićanin MD . Evaluation of staining‐dependent colour changes in resin composites using principal component analysis. Sci Rep. 2015;5:14638. 10.1038/srep14638 26450008 PMC4598812

[42] Wong J , Yeo C , The M , Taneski F , Josic U , Breschi L , et al. Are sculptable bulk‐fill composites susceptible to color change: a systematic review. J Esthet Restor Dent. 2025;38:70–85. 10.1111/jerd.70044 41069212

[43] de Deus RA , Oliveira L , Braga S , Ribeiro M , Price RB , Núñez A , et al. Effect of radiant exposure on the physical and mechanical properties of 10 flowable and high‐viscosity bulk‐fill resin composites. Oper Dent. 2024;49(2):136–56. 10.2341/23-025-L 38349819

[44] Gonçalves F , Campos LMP , Rodrigues‐Júnior EC , Costa FV , Marques PA , Francci CE , et al. A comparative study of bulk‐fill composites: degree of conversion, post‐gel shrinkage and cytotoxicity. Braz Oral Res. 2018;32:e17. 10.1590/1807-3107bor-2018.vol32.0017 29538479

[45] Bolaños‐Carmona V , Benavides‐Reyes C , González‐López S , González‐Rodríguez P , Álvarez‐Lloret P . Influence of spectroscopic techniques on the estimation of the degree of conversion of bulk‐fill composites. Oper Dent. 2020;45(1):92–103. 10.2341/18-095-L 31750799

